# Dr. Clement T. Guillaume

**Published:** 1917-07

**Authors:** 


**Dr. Clement T. Guillaume,** Eclectic of Cincinnati, 1880, of Boonville, died in Utica, May 10, aged 61.

